# Pneumoperitoneum Secondary to Necrotic Intra-abdominal Lymph Node

**DOI:** 10.7759/cureus.47871

**Published:** 2023-10-28

**Authors:** Nicholas S Cairl, Victoria L Sharp

**Affiliations:** 1 General Surgery, Trinity Health Ann Arbor Hospital, Ann Arbor, USA; 2 Trauma, Acute, and Critical Care Surgery, Trinity Health Ann Arbor Hospital, Ann Arbor, USA

**Keywords:** lymphoma, abdominal infection, general surgery, acute care surgery, pneumoperitoneum

## Abstract

Pneumoperitoneum is often treated as a surgical emergency as the most common etiology is perforated hollow viscus. Here, we present the case of a man in his 70s who presented to the emergency department with fever and abdominal pain. On physical exam, he was diffusely tender in the bilateral lower quadrants with guarding. Imaging demonstrated moderate volume pneumoperitoneum. On review of his imaging, the pneumoperitoneum was centered around a 7 cm necrotic lymph node. Repeat CT scan with positive oral (PO) and rectal contrast demonstrated no extraluminal contrast extravasation, but air bubbles were seen extending from the necrotic lymph node into the lower abdominal cavity. He underwent CT-guided drain placement and was started on antibiotics, and improved without surgical intervention. In stable patients presenting with pneumoperitoneum and known intra-abdominal lymphadenopathy, perforated viscus should be ruled out prior to surgical intervention, and necrotic intra-abdominal lymph node should be considered as a differential diagnosis.

## Introduction

Surgical dogma teaches that pneumoperitoneum, or air within the abdominal cavity, is a surgical emergency demanding operative exploration as the most common cause is perforated hollow viscus [1]. There are, though, several etiologies of pneumoperitoneum that do not require operative intervention. Retained post-operative air, peritoneal dialysis, recent endoscopy, and mechanical ventilation are causes of pneumoperitoneum that do not require operative exploration [2]. Due to the wide differential diagnosis and potential for surgical emergency, patients presenting with pneumoperitoneum should undergo surgical evaluation.

## Case presentation

**Figure 1:**
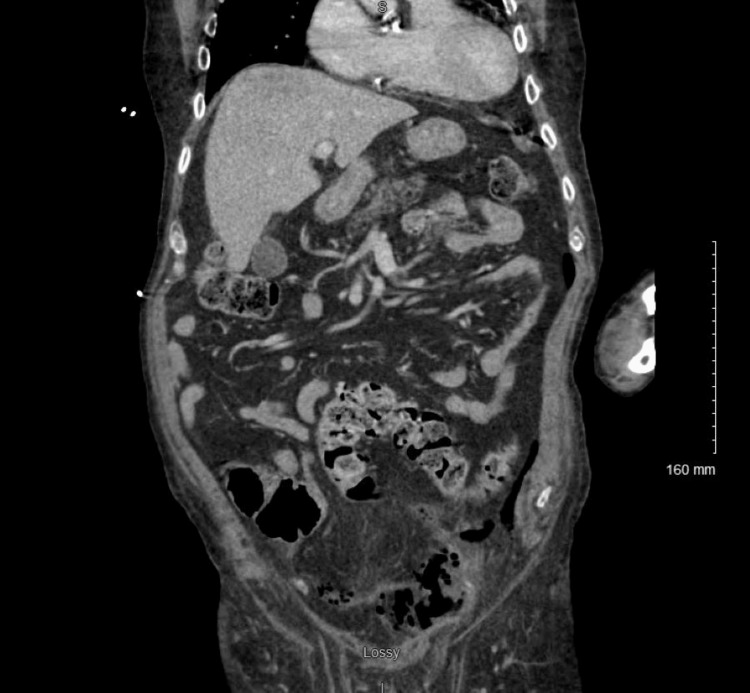
CT abdomen and pelvis with IV contrast demonstrating moderate pneumoperitoneum

A man in his 70s presented to the emergency department with several days of subjective fever and abdominal pain. His medical history was significant for diffuse B-cell lymphoma on chemotherapy, permanent atrial fibrillation on Eliquis, and recent admission for chemotherapy-induced pneumonitis, for which he was discharged on high-dose steroid taper. On physical exam, he was tender to palpation in the bilateral lower quadrants with guarding but without signs of peritonitis. Laboratory analysis was significant for a white blood cell count of 4.4 K/mcl. Computed tomography (CT) of the abdomen and pelvis with intravenous (IV) contrast demonstrated moderate volume pneumoperitoneum (Figure 1).

**Figure 2:**
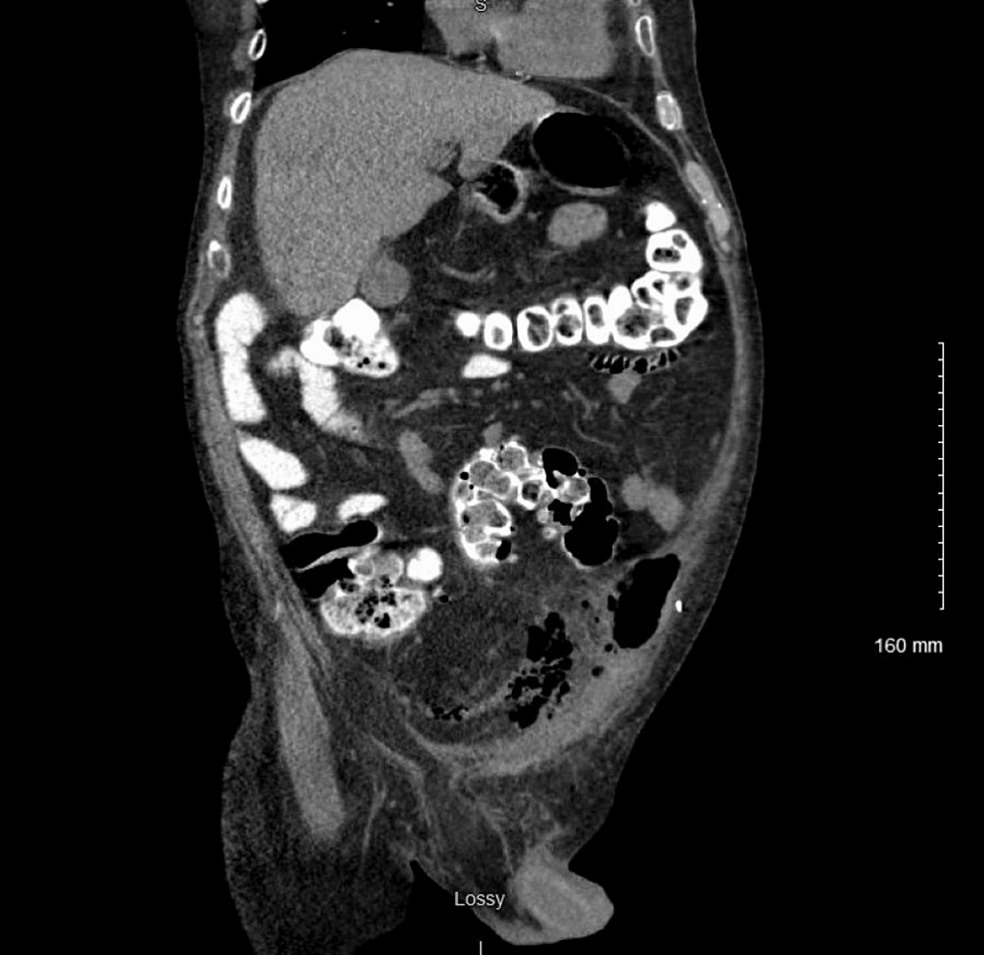
CT abdomen and pelvis with oral and rectal contrast demonstrating no contrast extravasation but air bubbles arising from a necrotic lymph node

On review of his imaging with radiology, the pneumoperitoneum was centered around a 7 cm necrotic lymph node identified on a CT scan of the abdomen and pelvis one month prior, which was completed to monitor response to chemotherapy. Given his hemodynamic stability and absence of peritonitis, a repeat CT scan of the abdomen and pelvis was obtained with oral and rectal contrast to rule out hollow viscus perforation. No extraluminal contrast extravasation was identified, but air bubbles were seen extending from the necrotic lymph node into the lower abdominal cavity (Figure 2).

The patient was admitted to the medicine service with interventional radiology and infectious disease consultation. The general surgery team continued to perform serial abdominal exams. On hospital day one, he underwent CT-guided percutaneous drain placement with return of thirty milliliters of purulent material. This was sent for culture and eventually grew *Enterobacter cloacae*. He was started on an extended course of cefepime per infectious disease team recommendations and was ultimately discharged to the inpatient rehabilitation unit on hospital day seven. 

## Discussion

Patients with pneumoperitoneum frequently present with abdominal pain and, depending on the source of the air, may also present with nausea, vomiting, fever, or sepsis [1]. Given the wide array of differential diagnoses, obtaining a pertinent history is important. Pneumoperitoneum is often identified on upright chest radiographs as air under the diaphragm, as this is often the first imaging test performed in the workup of patients presenting with acute abdominal pain. CT, though, is the most sensitive imaging study for the diagnosis of pneumoperitoneum [3]. The use of oral and rectal contrast can also be used to localize the potential perforation. Depending on the source of pneumoperitoneum or in cases of hemodynamic instability or peritonitis, operative intervention may be indicated; however, there are cases where non-operative management is appropriate.

In patients presenting with pneumoperitoneum, there should be a high index of clinical suspicion for perforated hollow viscus; however, there are causes of pneumoperitoneum that do not require surgical intervention. There are no reported cases of pneumoperitoneum secondary to necrotic intra-abdominal lymph nodes in the literature. Given this patient's multiple co-morbidities requiring Eliquis and high-dose steroids and his current chemotherapy treatment, operative intervention would have increased his risk of morbidity and mortality. Based on the American College of Surgeons surgical risk calculator, which uses patient-specific risk information using twenty patient predictors and the planned procedure to predict any of eighteen different outcomes within thirty days following surgery, undergoing exploratory laparotomy would have carried a 36% chance of any complication compared to an average risk of 20% and a 21% risk of mortality compared to an average risk of 4% [4].

## Conclusions

In stable patients presenting with pneumoperitoneum and known intra-abdominal lymphadenopathy, perforated viscus should be ruled out with oral and rectal contrast prior to surgical intervention, and necrotic intra-abdominal lymph node should be considered as a differential diagnosis.
